# Allogenic Fc Domain-Facilitated Uptake of IgG in Nasal *Lamina Propria*: Friend or Foe for Intranasal CNS Delivery?

**DOI:** 10.3390/pharmaceutics10030107

**Published:** 2018-07-26

**Authors:** Simone Ladel, Johannes Flamm, Arghavan Soleimani Zadeh, Dorothea Filzwieser, Julia-Christina Walter, Patrick Schlossbauer, Ralf Kinscherf, Katharina Lischka, Harald Luksch, Katharina Schindowski

**Affiliations:** 1 Institute of Applied Biotechnology, University of Applied Science Biberach, 88400 Biberach, Germany; ladel@hochschule-bc.de (S.L.); flamm@hochschule-bc.de (J.F.); soleimanizadeh@hochschule-bc.de or arghavan.soleimani@uni-ulm.de (A.S.Z.); dorothea.filzwieser@hochschule-bc.de (D.F.); juliawalter1993@yahoo.de or julia-christina.walter@uni-ulm.de (J.-C.W.); patrick.schlossbauer@hochschule-bc.de (P.S.); 2 Faculty for Natural Sciences, University of Ulm, 89081 Ulm, Germany; 3 Faculty of Medicine, Graduate School ‘Molecular Medicine’, University of Ulm, 89081 Ulm, Germany; 4 Department of Medical Cell Biology, Institute for Anatomy and Cell Biology, Philipps-University Marburg, 35032 Marburg, Germany; Ralf.Kinscherf@uni-marburg.de; 5 Chair of Zoology, Technical University of Munich, 85354 Freising-Weihenstephan, Germany; katharina.lischka@tum.de (K.L.); harald.luksch@wzw.tum.de (H.L.)

**Keywords:** olfactory epithelium, respiratory epithelium, nasal mucosa, NALT, lymphoid follicles, neuronal bundles, antibody, permeation, nose to brain, drug delivery

## Abstract

Background: The use of therapeutic antibodies for the treatment of neurological diseases is of increasing interest. Nose-to-brain drug delivery is one strategy to bypass the blood brain barrier. The neonatal Fc receptor (FcRn) plays an important role in transepithelial transcytosis of immunoglobulin G (IgG). Recently, the presence of the FcRn was observed in nasal respiratory mucosa. The aim of the present study was to determine the presence of functional FcRn in olfactory mucosa and to evaluate its role in drug delivery. Methods: Immunoreactivity and messenger RNA (mRNA) expression of FcRn was determined in ex vivo porcine olfactory mucosa. Uptake of IgG was performed in a side-by-side cell and analysed by immunofluorescence. Results: FcRn was found in epithelial and basal cells of the olfactory epithelium as well as in glands, cavernous bodies and blood vessels. Allogenic porcine IgGs were found time-dependently in the *lamina propria* and along axonal bundles, while only small amounts of xenogenic human IgGs were detected. Interestingly, lymphoid follicles were spared from allogenic IgGs. Conclusion: Fc-mediated transport of IgG across the nasal epithelial barrier may have significant potential for intranasal delivery, but the relevance of immune interaction in lymphoid follicles must be clarified to avoid immunogenicity.

## 1. Introduction

Biopharmaceuticals are of increasing importance in the therapy of various diseases. Since the development of the hybridoma technology by Köhler and Milstein, some of the most important biopharmaceutical molecules are antibodies, in particular immunoglobulin G (IgGs) [1,2].

Despite their great therapeutic potential, the tissue penetration of large molecules like IgGs is a critical aspect for the development of therapies and administration routes, in particular for neurological disorders. The blood-brain barrier (BBB) is a highly selective barrier and severely limits the use of antibodies for the therapy of diseases of the central nervous system (CNS). The BBB consists of cerebral vascular endothelial cells that are firmly connected by tight junctions and surrounded by astroglia expressing soluble factors responsible for the formation of tight junctions [3,4,5].

One strategy to bypass the BBB is to deliver drugs to the CNS by intranasal drug delivery. In humans intranasally administered insulin was already shown to have a positive effect on memory and metabolic effects via the hypothalamic-pituitary-axis [6,7,8,9,10,11]. Advantages of the intranasal route are the minimally-invasive administration, reduced systemic side effects due to direct CNS targeting, and improved patient compliance compared to intrathecal delivery [6,7]. As the feasibility of nose to brain (N2B) drug delivery was already shown for sumatriptan, oxytocin, insulin, and some other drugs, it is of particular interest to investigate whether the N2B route is also suitable for proteins with a higher molecular weight such as antibodies [7,12,13,14].

IgG antibodies contain two different kinds of polypeptide chains: the heavy chain and light chain. Each IgG is composed of two heavy chains that are linked via disulfide bond as well as two light chains that are respectively connected by disulfide bonds. The light chain consists of one variable domain (V_L_) and one constant domain (C_L_). The heavy chain is structured into four domains: three constant domains (C_H_1, C_H_2, C_H_3) and one variable domain (V_H_) [15,16].

**Figure 1:**
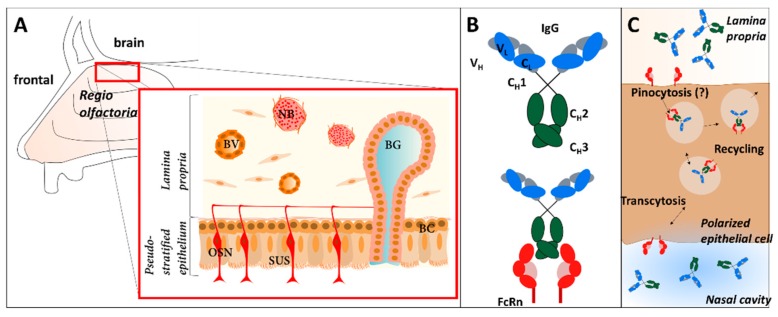
Transcytosis and recycling of IgGs in the nasal mucosa mediated by the neonatal Fc receptor (FcRn) and structural overview of the mucosa composition. (**A**) The olfactory mucosa in mammals is composed of a pseudostratified epithelium that contains olfactory sensory neurons (OSN), supporting cells (SUS) and basal cells. The olfactory epithelium is lined by a thick connective tissue, called the *lamina propria*, containing Bowman’s gland (BG), blood vessels (BV), and neuronal bundles (NB). (**B**) Structure of immunoglobulin G (IgG) and binding to FcRn. (**C**) Immunoglobulin G (IgG) is incorporated into polarized cells of the epithelium via pinocytosis. The FcRn binds to the Fc-domain of the antibody in the slightly acidic environment of the early endosome and mediates transcytosis to the basolateral side or recycling of the IgG to the apical side. Transcytosis is shown here from apical (nasal cavity) to basolateral, but the reverse direction was also reported.

In endothelial cells and monocytes but also in epithelial cells, a specialized IgG transporter, the neonatal Fc receptor (FcRn) binds specifically to the C_H_3 and parts of the C_H_2 regions of IgGs (Figure 1C). The FcRn is composed of a heterodimer of major immunohistocompatability (MHC) class I-like heavy chain and a microglobulin β light chain. It binds the Fcγ domain preferentially at pH < 6.5 but hardly at physiological pH [17,18,19]. FcRn immunoreactivity was previously demonstrated by Heidl et al. in the human nasal respiratory mucosa of the inferior turbinate, in particular in epithelial, basal, endothelial and gland cells. However, the presence of FcRn in the *regio olfactoria* of higher mammals, a region that is highly implicated in N2B drug delivery, has yet not been described [19,20,21].

As demonstrated for lung and gut, FcRn can facilitate IgG transport from the apical to the basolateral side in polarized cells and vice versa [22,23]. As indicated in Figure 1B,C, IgGs are taken up by pinocytosis, but also specific uptake via FcRn is discussed [24,25,26]. In the acidic environment of the early endosome, the Fcγ domain binds to the FcRn. During exocytosis, after recycling or transcytosis, the pH shift at the extracellular environment to physiological pH (pH 7.4) causes a release of the IgG molecule [27].

Ober et al. showed cross-species transport of human IgG by the porcine FcRn, making porcine tissue a promising model organism to test ex vivo epithelial transport of IgGs [28]. In addition to the molecular similarities, the cellular composition of the respiratory mucosa was also shown to be comparable between humans and pigs [29,30]. Thus, it is reasonable to assume that there are strong similarities between human and porcine olfactory mucosa. The olfactory mucosa in general is composed of a pseudostratified epithelium containing polarized epithelial cells, olfactory neurons, basal cells (progenitor cells), and supporting cells. Beneath the basal cell layer, there is a thick layer of connective tissue with neuronal bundles, Bowman’s glands, cavernous bodies and smaller blood capillaries (Figure 1A) [31,32,33]. This layer is called the *lamina propria*. In addition, Debertin and colleagues investigated the nasal immune system in newborn children. They found B and T lymphocytes arranged in so-called lymphoid follicles similar to the Peyer’s patches in the gut [34].

The neuronal bundles are accumulated axons of the olfactory receptor neurons projecting to the olfactory bulb, a brain region specialized for olfaction [35]. Balin et al. demonstrated that horseradish peroxidase (HRP), administered to the olfactory mucosa, was detectable in the olfactory bulb of rodents and monkeys within 45 to 90 min [36]. Therefore, it could be assumed that axons of the olfactory neurons present a pathway into the brain for therapeutic protein drugs such as antibodies [37].

In a previous study, the permeation of the human therapeutic IgG Avastin^®^ (bevacizumab) through two subtypes of porcine mucosa from the septum and the snout cavity was shown, and a link between FcRn and the IgG’s pathway through the epithelium was suspected [38].

The aim of the present ex vivo study was to evaluate the presence of FcRn in porcine olfactory mucosa. Nasal porcine mucosa explants were excised from the dorsal part of the *concha nasalis dorsalis* (superior turbinate in humans) covered with olfactory mucosa and from the *concha nasalis ventralis* (inferior turbinate in humans) that is covered with respiratory mucosa. Furthermore, the function of FcRn was evaluated by determining the qualitative transport of allogenic porcine IgGs in comparison to a xenogenic human IgG (a biosimilar of bevacizumab). These experiments should also clarify whether xenogenic human IgGs are transported via the porcine FcRn. Using immunofluorescence, qualitative uptake and the fate of IgGs in the *lamina propria* was investigated, in particular in neuronal bundles and in lymphoid follicles.

## 2. Material and Methods

### 2.1. Antibodies 

According to Table 1 the following antibodies were used for uptake and distribution studies as well as for immunofluorescence and Western blotting.

### 2.2. Tissue Preparation

The mucosa explants were collected from the nasal cavity of slaughterhouse pigs aged 4 to 6 months old from a local butcher. The tissue specimens were excised from the dorsal part of the *concha nasalis dorsalis* (olfactory epithelium) and the middle part of the *concha nasalis ventralis* (respiratory epithelium) according to [39]. In detail, approximately 2 cm^2^ of the mucosa were dissected with a scalpel and removed gently from the cartilage using a blunt spatula to avoid damage to the mucosa explants. The post mortem delay of the porcine tissue was below 2 h. For morphological comparison human *regio olfactoria* was excised from anatomical donations fixed in 4% paraformaldehyde/96% ethanol for anatomical teaching courses. The human specimens were used including cartilages as the tissue was too fragile to remove the mucosa without damage. The different qualities observed in the sections from human and porcine tissue are due to these different tissue processing procedures.

### 2.3. Reverse Transcription and Polymerase Chain Reaction (PCR)

To isolate total RNA from the specimens, TRIzol (Thermo Fisher Scientific, Dreieich, Germany) was used according to the manufacturer’s instructions. Tissue sections of 200 mg were used per library and the RNA was stored at −80 °C. For reverse transcription to complementary DNA (cDNA), 1 µg of total RNA was mixed with 2U RNAse inhibitor (Invitrogen^TM^, USA) and added up to 10 µL with ultra-pure distilled RNAse-free water (Invitrogen™, USA). The RNA secondary structure was denatured by heating to 65 °C for 15 min. Per reaction, 100 pM oligo-dT_15_ primer, 20 mM deoxynucleotides (dNTPs), and 400 U murine leukemia virus (MLV) reverse transcriptase were diluted in M-MLV buffer containing ultra-pure distilled RNase-free water and were added to the denatured RNA. The mix was incubated at 37 °C for 1 h and then inactivated for 10 min at 65 °C. The cDNA templates were stored at −20 °C until use.

2 µg cDNA, 1 µM of the appropriate primer pairs (see Table 2; Thermo Fisher Scientific, Dreieich, Germany), 25 mM MgCl_2_ (Thermo Fisher Scientific, Dreieich, Germany), 2.5 mM dNTP Mix (Thermo Fisher Scientific, Dreieich, Germany), and 0.5 U/µL Taq polymerase (Invitrogen™, USA) were diluted in Taq-PCR buffer (Thermo Fisher Scientific, Dreieich, Germany) containing RNAse-free water (Invitrogen™, USA) to amplify the DNA target sequences by PCR.

The PCR was performed with initial denaturation for 30 s at 95 °C, 40 cycles with denaturation at 95 °C for 30 s, annealing at 60 °C for 30 s, elongation at 72 °C for 60 s, and a final elongation at 72 °C for 10 min. The amplicons were analysed by agarose gel electrophoresis.

### 2.4. Western Blot

The tissue explants were homogenized, chilled RIPA (radioimmunoprecipitation) cell lysis buffer (10 mM Tris-Cl, pH8.0; 1 mM EDTA, 0.5 mM EGTA, 1% Triton X-100, 0.1% sodium deoxycholate, 0.1% SDS (sodium dodecyl sulfate), 140 mM NaCl, and protease inhibitor mix (Thermo Fisher Scientific, Dreieich, Germany)) were added, and the samples were agitated for complete cell lysis.

Equal volumes of homogenized tissue were loaded, separated in a 12.5% SDS PAGE, and blotted onto a nitrocellulose membrane (Carl Roth, Karlsruhe, Germany). The membrane was blocked (5% skimmed milk powder in PBS/0.1% Tween20, pH 7.4). Primary antibodies (details see Table 1) were diluted by 1:5000 and incubated overnight at 4 °C. The secondary antibodies were used at 1:100,000 (Anti-rabbit IgG-HRP) and 1:4000 (Anti-murine IgG-HRP), and the membrane developed with the chemoluminescence substrate Immobilon^®^ (Merck Millipore, Darmstadt, Germany) according to the manufacturer’s instructions. Image acquisition and quantitation of band intensity were performed using Fusion FX Imaging systems (VILBER Lourmat, Collégien, France) and Image J (java.version: 1.8.0_171).

### 2.5. Uptake and Distribution Studies

**Figure 2:**
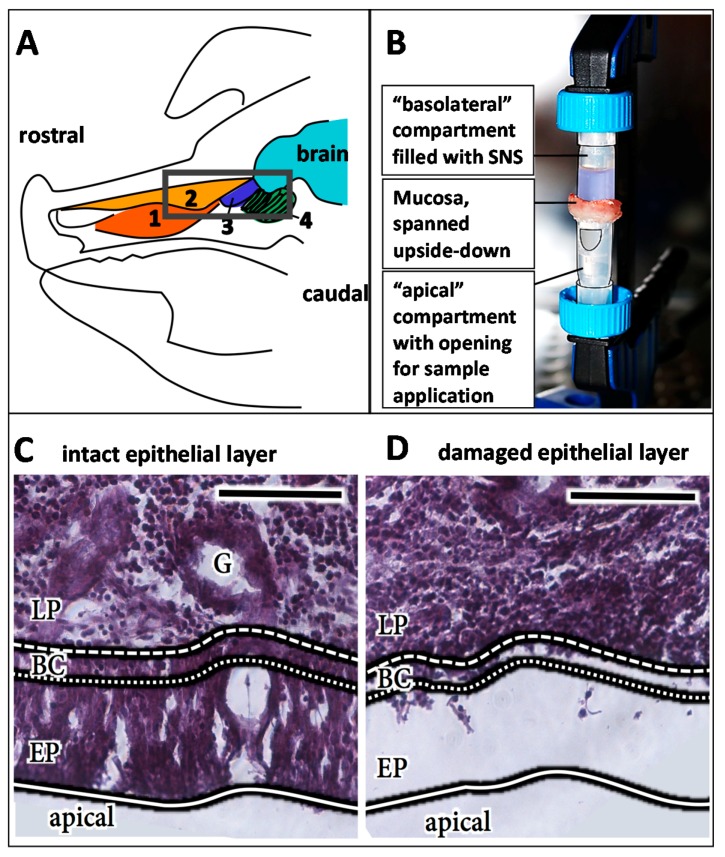
Experimental set-up of the uptake and distribution studies. Anatomical sketch of the sagittal section of a pig head according to [39], (**A**) 1: *concha nasalis ventralis*; 2: *concha nasalis dorsalis*; 3: *concha nasalis media*; 4: ethmoidal turbinates; Black box: *regio olfactoria*, from which tissue specimens were taken. (**B**) Experimental set-up: The mucosa specimen was fixed with a fastening clamp upside down in between two microreaction tubes. In the lower tube, a small opening was cut into the tube wall to obtain access to the mucosal surface for antibody or vehicle (PBS) application. (**C**) HE staining of an intact epithelial layer. (**D**) HE staining of a damaged epithelial layer. Detachment and damage of the epithelial layer leads to loss of its barrier function and, hence, has an important influence on the experimental read-out (see also Supplementary Materials). Therefore, all samples were analysed for the integrity of their epithelial layer by HE staining, and damaged samples as shown here were excluded from the analysis. LP: *lamina propria*; BC: basal cells; EP: epithelial layer. Scale bar: 100 µm.

To simulate the upside-down conditions at the olfactory region, a 2 cm^2^ mucosa specimen was placed in a modified side-by-side cell consisting of two microreaction tubes (1.5 mL) to avoid leakages (Figure 2B). 1 mL of simulated nasal solution (SNS, 1.5 mM NaH_2_PO_4_, 0.83 mM NaHPO_4_, 1.67 mM Mg_2_Cl_2_, 4.56 mM KCl, 119.78 mM NaCl, 10 mM d-glucose, 15 mM NaHCO_3_, 1.2 mM CaCl_2_, osmolarity: 300 ± 10 mol/kg; pH 7.4; buffer was oxygenated for 10 min before use [40]) was filled in the upper tube prior to sealing with the mucosa. The assay set-up was developed to display conditions as similar as possible to in vivo conditions. Therefore, the mucosa explants were not washed before placing them in the side-by-side cell, and the mucus layer was not removed to keep the system as close as possible to the native situation in intranasal drug application (mucus, upside-down, temperature: 35 °C).

A volume of 10 µL containing 8 mg/mL (54 µM) porcine serum IgGs in 0.1 M phosphate-buffered saline (PBS, Sigma Aldrich, Taufkirchen, Germany) or the same concentration of the humanized antibody bevacizumab biosimilar (in-house production) were pipetted carefully onto the epithelial layer. The mucosa was incubated at 35 °C and >90% humidity for either 30 min, 2 h, 4 h, or 8 h. The negative/vehicle control (buffer without antibody) was incubated for 8 h in parallel. At least four independent experiments were performed, and representative data are shown. Samples without an intact epithelial layer (see below) were excluded, and the experiment was repeated to obtain the necessary number of intact samples.

After the respective incubation time, the mucosa explants were directly fixed in 4% paraformaldehyde for 2 h, cryoconserved in 30% sucrose overnight, and stored at 4 °C until sectioning. The tissue was cut in 14 µm slices in a cryostat at −25 °C (HM525 NX, Thermo Fisher Scientific, Dreieich, Germany) and mounted on Superfrost^®^Plus Micro slides (VWR International GmbH, Darmstadt, Germany).

### 2.6. Immunohistochemistry and Histological Staining

Slides were washed three times for 5 min with PBS (Roti®fair PBS 7.2, Carl Roth, Karlsruhe, Germandy; pH 7.4) followed by blocking with 4% BSA, 0.5% Saponin and 10% NGS solution in PBS for 1 h. The primary antibodies (Table 1) were diluted 1:100 in PBS containing 4% BSA/0.5% Saponin and incubated on the tissue sections for 2 days at 4 °C. The slices were washed again (5 min, 10 min, and 15 min), and incubated with the corresponding secondary antibodies (Table 1, 1:500 diluted in PBS containing 4% BSA/1% Saponin) for 2 h. After another three washing steps as described before, slides were mounted with Fluoroshield™ mounting medium containing DAPI (4′,6-diamidin-2-phenylindol; Sigma-Aldrich, Taufkirchen, Germany).

To verify tissue integrity, adjacent or following slides from each sample were stained with hematoxylin-eosin (HE; Gil III, Merck Millipore, Germany). Briefly, the slides were washed with distilled water for 1 min, followed by staining in hematoxylin solution for 2 min, destained under running tap water for 12 min, and counterstained with Eosin-0.5% acidic acid (Sigma-Aldrich, Taufkirchen, Germany) for 3 min. Slides were additionally destained under running tap water for 1 min. Subsequently, the slides were dehydrated and mounted in Eukitt Quick hardening mounting medium (Sigma-Aldrich, Taufkirchen, Germany). The human tissue sections were only HE-stained similar to the porcine sections.

### 2.7. Analysis

The samples were analysed either by phase-contrast microscopy (NikonEclipse 80 I; Nikon Instruments Europe B.V., Duesseldorf, Germany) and epi-fluorescence microscopy (Olympus BX63, Olympus Europa SE & Co. KG, Hamburg, Germany) or by confocal microscopy (Zeiss LSM 7MP, Carl Zeiss AG, Jena, Germany). To blank the signal of endogenous porcine IgGs from the exogenously administered allogenic porcine serum IgGs (Sigma-Aldrich, Taufkirchen, Germany), the Image J (java.version: 1.8.0_171) macro “Image Calculator” was used. With this tool, the fluorescence intensity of the negative/vehicle control stained with anti-swine-RhodamineRed secondary antibody was subtracted from the respective sample tif data files. For comparability, the FITC signal of hIgG was converted to a red colour in Image J.

## 3. Results

The bioavailability in the CNS of intravenously administrated antibodies is limited and N2B drug delivery is discussed as an attractive alternative, in particular for higher molecular weight biopharmaceuticals. In this context, the antibody transporter FcRn is implicated in transport and distribution of IgGs from mucosal surfaces. Thus, the aim of the present study was to evaluate the presence and the function of FcRn in porcine olfactory mucosa explants and finally the fate of transported IgGs in the nasal *lamina propria*. The presence of FcRn could facilitate IgG intracellular uptake from endosomes and tissue distribution, but FcRn is also associated in mucosal immune defence and, thus, FcRn interaction with biopharmaceuticals could result in undesired immunogenicity.

**Figure 3:**
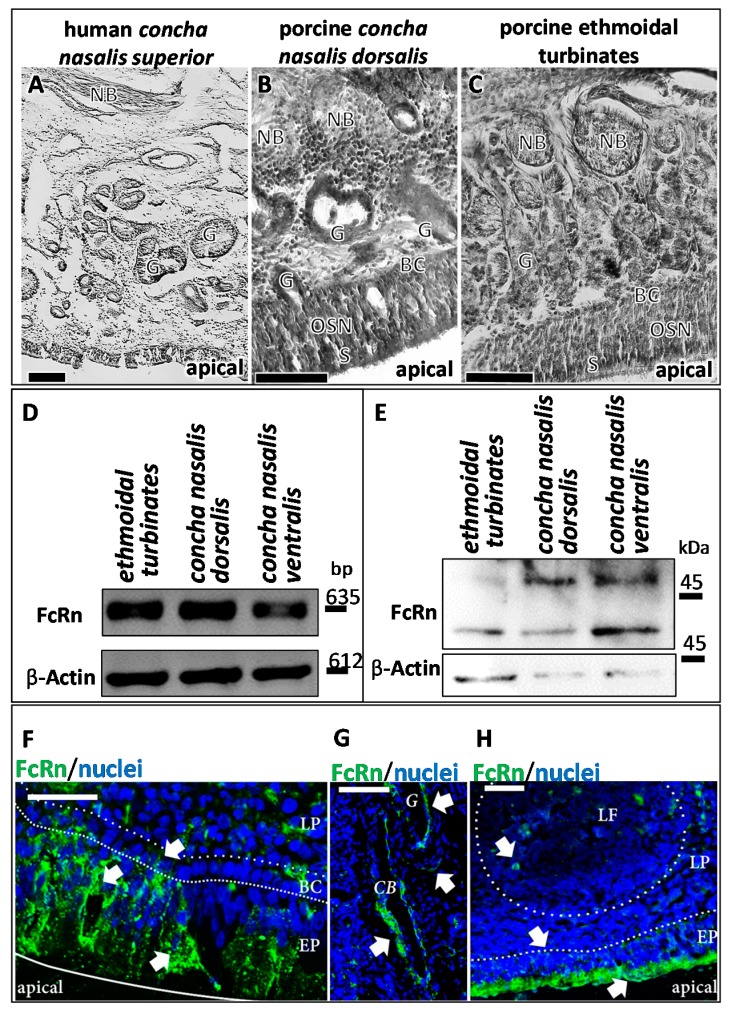
FcRn in porcine olfactory mucosa. (**A**–**C**) Porcine olfactory mucosa shows a similar architecture as observed in humans [44]. Comparable to human tissue (**A**); neuronal bundles, Bowman’s glands and a pseudostratified epithelium are found in the porcine *concha nasalis dorsalis* (**B**); and in the porcine ethmoidal turbinates (**C**); Scale bar: 200 µm. FcRn expression and immunoreactivity were evaluated in the *regio olfactoria* by reverse transcriptase-PCR (RT-PCR) (**D**) and Western Blot (**E**). Protein (**E**) and mRNA (**D**) of FcRn were observed in all investigated olfactory regions: Ethmoidal turbinates (*regio olfactoria*), *concha nasalis ventralis* (respiratory region), *concha nasalis media* (*regio olfactoria*, not shown), and *concha nasalis dorsalis*. PCR fragment sizes: FcRn:635 bp; Beta Actin: 612 bp (loading control). Molecular Weight: FcRn:40 kDa; higher molecular weight band FcRn: different glycosylation patterns according to [17]; Beta Actin: 42 kDa (loading control). FcRn immunoreactivity was observed throughout the porcine olfactory mucosa: (**F**) FcRn is detected in epithelial cells, cells of the basal membrane and in blood vessels. In epithelial cells, FcRn is observed in vesicles, but apparently also at the apical side; Scale bar: 50 µm. (**G**): FcRn expression in cavernous bodies and glands in the *lamina propria*; Scale bar: 50 µm. (**H**): vesicular transport of FcRn in the epithelial layer and FcRn expression in lymphoid follicles; Scale bar: 200 µm; DAPI stained nuclei. BC: basal cells; CB: cavernous body; G: glands; LF: lymphoid follicle; LP: *lamina propria*; NB: neuronal bundles; OSN: olfactory sensory neurons; SUS: sustentacular cells.

The use of appropriate human nasal tissue is ethically challenging and mostly limited to specimens from nasal surgery, which are predominantly from polyps or lower parts of the nasal turbinates that are covered with respiratory mucosa only. Therefore, fresh porcine nasal tissue from the roof of the nasal cavity containing predominantly olfactory mucosa was used here. Comparison of the morphology of the *regio olfactoria* from humans and pigs showed a high similarity (Figure 3A), and these findings are supported by published data [41,42,43]. The use of porcine tissue as a model to determine the penetration and distribution of antibodies, both allogenic and xenogenic, i.e., porcine (pIgG) and human IgGs (hIgG) was investigated in this study.

### 3.1. Presence and Localisation of FcRn in the Porcine Regio Olfactoria

The key histological features of the olfactory mucosa include an epithelium with basal cells, olfactory sensory neurons, and sustentacular cells, as well as axonal bundles and glands with epithelial openings (Bowman’s glands) in the *lamina propria* [44]. All these characteristics were found in the porcine *regio olfactoria*, especially in the dorsal part of the *concha nasalis media (not shown)*, the dorsal part of the *concha nasalis dorsalis*, and the *ethmoidal turbinates* (Figure 3A–C).

In humans, Heidl et al. demonstrated immunoreactivity against FcRn in epithelial cells, basal cells, gland cells, and endothelial cells of the nasal mucosa [19]. In line with these findings, FcRn expression was confirmed by RT-PCR and Western Blot in different parts of the porcine *regio olfactoria* (*concha nasalis dorsalis, concha nasalis media, ethmoidal turbinates*) as well as in the porcine respiratory epithelium (*concha nasalis ventralis*; Figure 3D,E).

Immunoreactivity against FcRn was observed in the porcine *regio olfactoria*, in particular in the epithelial cells, mainly at the apical sides, but also inside their cell bodies. Furthermore, immunoreactivity was revealed at both, the apical and basolateral sides of cavernous bodies and at the apical side of gland cells as wells, as at the apical side of endothelial cells forming blood vessels (Figure 3F–H).

### 3.2. IgG Uptake and Distribution in Porcine Olfactory Mucosa Explants

**Figure 4:**
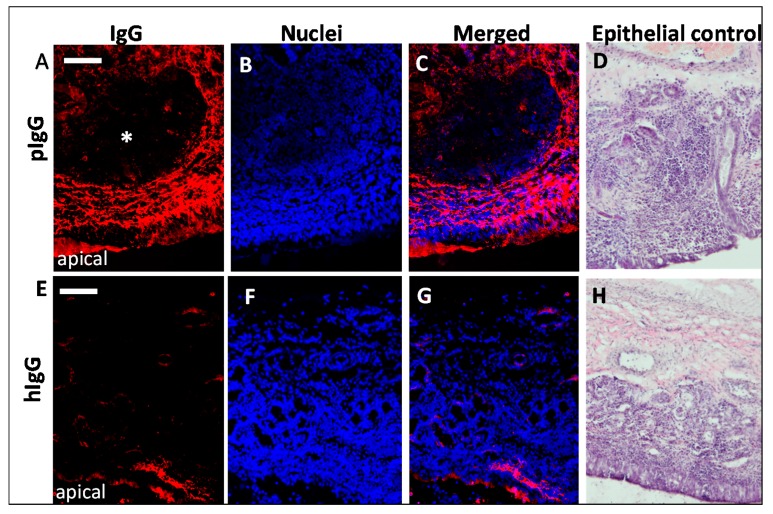
Comparison of uptake and distribution of porcine vs. human IgG through porcine olfactory mucosa. (**A**,**C**) immunoreactivity against porcine IgG (pIgG) after 8 h incubation and (**E**,**G**) immunoreactivity against human IgG (hIgG; bevacizumab biosimilar) after 8 h incubation (intrinsic porcine IgG signal was subtracted for all data presented). (**B**,**F**): nuclei counterstain; (**C**): Image of pIgG staining and nuclei overlay after 8 h incubation. (**D**,**H**) HE staining of the mucosa explants as quality control for an intact epithelial barrier. Moreover, round subepithelial structures were observed (*) that seem to be spared from IgG immunoreactivity. Nuclei are stained with DAPI; Scale bar: 200 µm.

The findings of an earlier study suggested the feasibility of porcine tissue for studying FcRn-mediated transport processes of human antibodies [28]. To investigate this assumption, allogenic porcine IgGs (pIgG) and xenogenic human monoclonal IgGs (hIgG) were applied to the apical side of olfactory mucosa in a side-by-side cell setup. The mucosa explants were collected after different incubation periods, fixed immediately, and processed for the immunofluorescent detection of IgG and FcRn. The mucosa received nutrients and oxygen via an oxygenated buffer at the basolateral side. In addition, it was observed by histology during the establishment of the method that even the smallest damages and injuries resulted in a loss of the epithelial layer. Any disruption of the epithelial layer during the preparation procedure destroyed its barrier function and resulted in complete penetration of the lamina propria by the applied IgGs after only 30 min (see Supplementary Figure S1). Therefore, the presence of an intact epithelial layer was confirmed as a quality control parameter for a successful experiment. Adjacent or following sections of all processed samples were used for HE staining to confirm that the epithelium was present and intact during the experiment (epithelial control, Figure 4).

To reduce the background caused by the basal endogenous levels of pIgG, vehicle-treated samples were processed identically and their detected fluorescence was subtracted from the IgG-treated samples. Both pIgGs as well as hIgGs penetrated the *lamina propria*, but with different kinetics and at significantly different levels. An obvious immunoreactivity against pIgG was observed after 8 h (Figure 4A–C) while the detected fluorescence indicative for immunoreactivity against hIgG was considerably lower (Figure 4E–G) after 8 h, but detectable in epithelial and basal cells. These data clearly implicate species-specific dependency of the transport. Interestingly, hIgG was also observed in cavernous bodies and blood vessels with a more diffuse pattern. In particular, the selective presence of bevacizumab at structures formed from endothelial cells may result from binding to its antigen vascular endothelial growth factor (VEGF). An alignment of human (UniProt ID P15692) and porcine (UniProt ID 49151) VEGFA revealed a 100% homology in the bevacizumab epitope from amino acid (aa)79 to aa94 [45]. Hence, it can be assumed that the localization of bevacizumab at blood vessels and cavernous bodies is related to binding of its antigen VEGF, which is secreted from endothelial cells.

### 3.3. Fc-Mediated Transport of Allogenic IgGs, but also Potential Fc-Mediated Clearance Pathways

**Figure 5:**
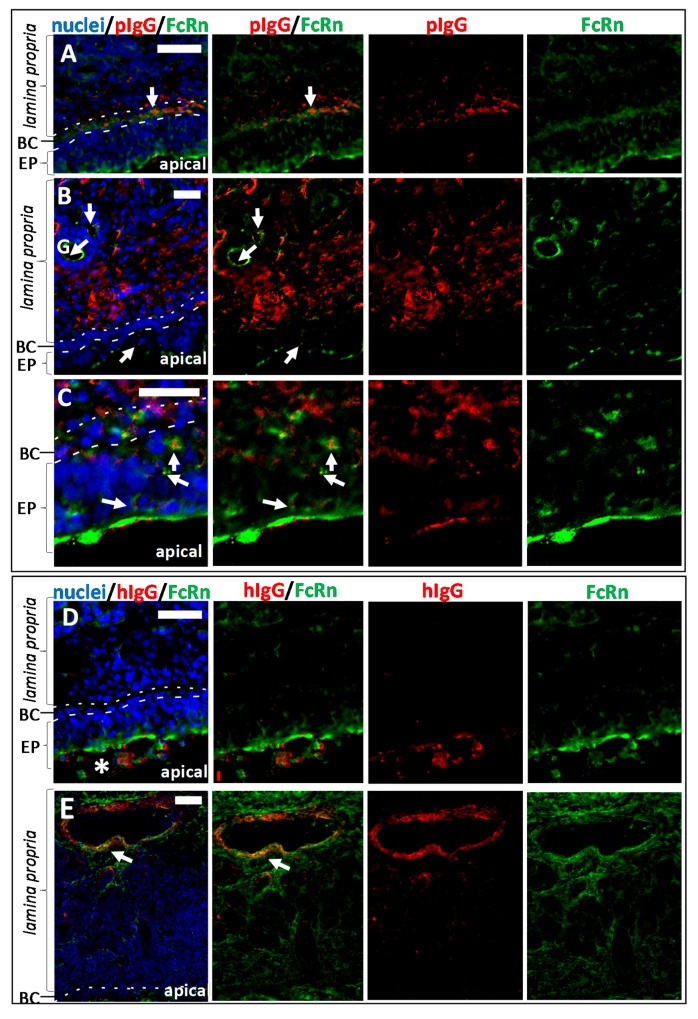
Co-localisation study of immunoreactivity against FcRn and IgG in the porcine *regio olfactoria*. (**A**) pIgG and FcRn localisation in the olfactory mucosa after 30 min of incubation. Co-localisation could already be detected in basal cells of the epithelial layer. (**B**) pIgG and FcRn localisation in the olfactory mucosa after 8 h of incubation. Double staining of FcRn and pIgG revealed co-localisation in glands (identified according to their morphology with an accumulation of round vessel-like structures), basal cells and polarized epithelial cells. (**C**) pIgG and FcRn co-localisation in the epithelial layer after 8 h of incubation. (**D**) Co-localisation of FcRn and hIgG (bevacizumab) after 0.5 h of incubation. The hIgG remains mainly at the apical surface (*). Diffusion through the epithelial layer was not detected after this incubation time. (**E**) After 8 h of hIgG incubation, co-localisation of FcRn and hIgG was mainly found in blood vessels and cavernous bodies, presumably caused by the presence of VEGF -the antigen of bevacizumab. Thus, in general the hIgG penetration into the tissue was weaker compared to pIgG. Arrows indicate sites of co-localisation. BC: basal cells; EP: epithelial layer; G: gland; Scale bar: 50 µm.

Earlier studies imply pinocytosis as an uptake mechanism of IgGs from the apical epithelial side, followed from fusion of early pinocytotic endosomes with FcRn-containing acidic endosomes. FcRn binds to IgGs and directs them to the basolateral side of the epithelium facing the *lamina propria*. Some other studies describe apical uptake via FcRn at low pH and transcytosis through the epithelial cells [24,25]. Regardless of the mechanism involved here, an indication for IgG-FcRn interactions should be observable by co-localisation studies. Thus, samples of pIgG, hIgG and vehicle experiments were stained for FcRn and pIgG, hIgG, respectively. The representative data in Figure 5 show co-localisation of FcRn and pIgG after 30 min of incubation, but hardly any co-localisation of FcRn and hIgG after the same incubation time. A weak co-localisation of hIgG with basal and epithelial cells was found at later incubation intervals. After 8 h, the above-mentioned diffuse signal was observed in blood vessels and cavernous bodies of the *lamina propia*. For pIgGs, clear co-localisation was found in blood vessels, cavernous bodies, glands, and the epithelial cells, including the basal cells (Figure 5B,C). Most probably due to fixation at the end of each experiment, the exogenously applied proteins could not be washed away and were still detectable (Figure 5C,D). Interestingly, only traces of pIgG were detected at the apical side, implying higher absorption. In contrast, large amounts of hIgG were apparently not taken up and remained at the apical side (see * in Figure 5D). The potential FcRn-mediated transport of epithelial and basal cells towards the *lamina propria* facilitated the apical uptake of allogenic, and in lesser amounts, xenogenic IgG. However, drainage to blood vessels could result in distribution to different compartments such as blood, and result in a reduction of the drug that is transported to the brain. Similarly, uptake of IgG by acinar cells of glands may lead to excretion of these IgGs with nasal mucus.

### 3.4. Kinetics of Allogenic IgG Uptake and Distribution into Olfactory Mucosa Lamina Propria

**Figure 6:**
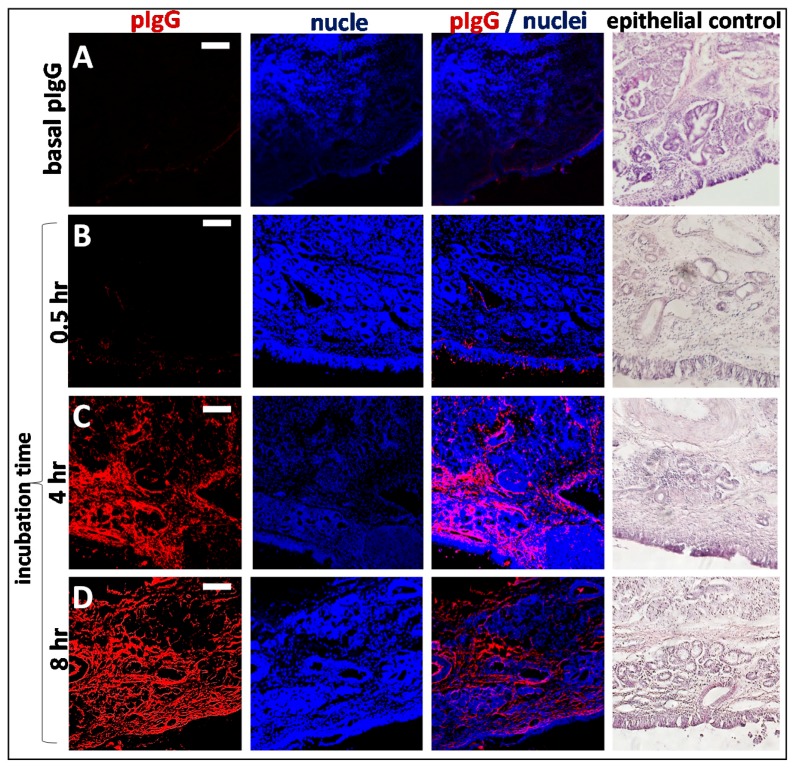
Time dependent penetration of pIgG through olfactory mucosa (*concha nasalis dorsalis*). (**A**) Basal levels of endogenous pIgG were detected with a low signal at the apical side, in the basal cell layer, glands, cavernous bodies, and blood vessels. This signal served as a blank and was subtracted from the photos showing the penetration of exogenous pIgG. (**B**) After 30 min, only the areas close to the apical side show immunoreactivity for pIgG, but some signal was detected in the *lamina propria*. (**C**) After 4 h, the pIgGs obviously distributed into the *lamina propria*. * indicate round structure filled with cells and mostly spared from IgG (**D**) After 8 h, pIgG were detected throughout the whole *lamina propria*. Nuclei stained with DAPI; epithelial control: quality control for tissue integrity, stained with HE. Scale bar: 100 µm.

A kinetic study of pIgG uptake in olfactory mucosa showed a time-dependent increase of IgG-immunoreactivity. While after 30 min only traces of pIgG were detectable within the epithelial cell layer (Figure 6), 2 h of incubation were sufficient to transport the pIgG to the *lamina propria* (data not shown). After 4 h of incubation, a clear strong immunofluorescent signal was detected throughout the whole *lamina propria*. This signal was only slightly amplified after 4 additional hours (8 h in total). As a concentration of 54 µM pIgG was used, it was assumed that all FcRn-dependent transport was saturated, thereby subsequently limiting the IgG’s transport velocity.

After 4 h and later, an interesting observation was obvious in most samples: large structures were spared more and more from pIgG immunoreactivity (*, Figure 4A–C; Figure 6). HE stain revealed these structures to be lymphoid follicles—nasal mucosa immune structures, which are similarly organized as intestinal Peyer’s patches [33,43,46].

As indicated above, the integrity of the epithelial layer was evaluated for all samples (Figure 6 last column). The result of an experiment with a damaged epithelium is shown in Supplementary Figure S1. Here, even at the earliest time investigated (30 min of incubation), a complete coverage of the *lamina propria* with the apically applied IgGs was observed.

### 3.5. The Fate of Allogenic IgG in the Lamina Propria and Potential Drug Routes to the Brain

**Figure 7:**
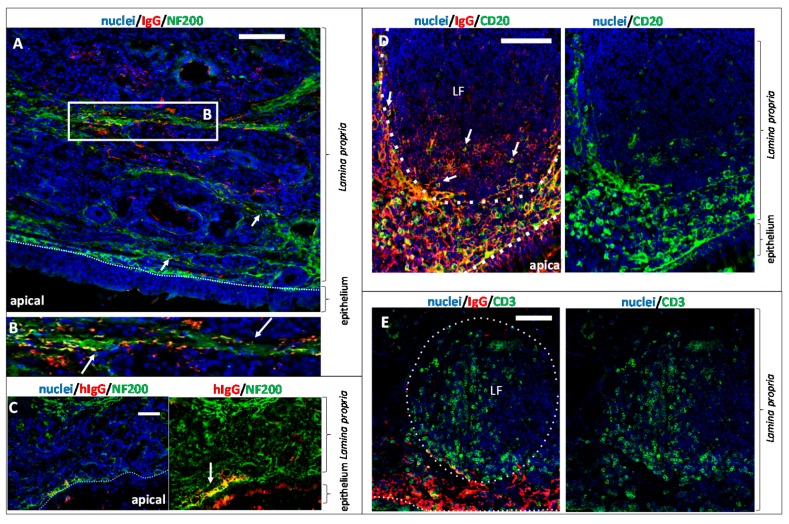
Exogenous IgG distribution in lymphoid follicles and potential interaction with neuronal fibres and the local immune system in the *regio olfactoria*. (**A**) 30 min of pIgG permeation: investigation of neuronal transport routes. The axonal marker Neurofilament 200 (NF200) was stained in green. pIgGs are stained in red. Arrowheads indicate pIgGs in close vicinity or co-localizing with neuronal fibres. pIgG in or along neuronal fibres were observed throughout the whole *lamina propria*, even in regions rather far away from the apical side. (**B**) Close-up of framed region in A. Co-localisation of NF200 and pIgG; Arrows indicate sites of co-localisation. (**C**) 8 h after apical hIgG application: co-localisation of hIgG and NF200 indicating a transport along the neuronal fibres. Note that hIgG was found in fibres, which are close to the apical site (**D**) Investigation of pIgG co-localizing with B cells of the lymphoid follicle after 8 h of incubation. B lymphocytes (antigen CD20) are stained in green. pIgGs are stained in red. (**E**) Lymphoid follicle in the *lamina propria* of the olfactory mucosa. Left: pIgG after 8 h of incubation (red), T lymphocytes (green), nuclei (DAPI, blue). LF: lymphoid follicle; Scale bar: 100 µm.

In N2B drug delivery, the neuronal fibres of the olfactory sensory neurons, but also the trigeminal nerve endings are of high interest as a potential transport route to the CNS [25]. The neuronal bundles travel through the cribriform plate to the olfactory bulb. Beneath the basal cell layer, olfactory ensheating cells enwrap neuronal bundles and are involved in neurogenesis of olfactory sensory neurons [47]. During tissue preparation, these projections have to be carefully disconnected with a blunt scalpel to excise the mucosa. Therefore, the whole potential route cannot be evaluated in this ex vivo model. Nevertheless, to elucidate uptake of pIgG either into olfactory axons or among the axons and the olfactory ensheating cells, double staining of the axonal marker neurofilament 200 (NF200, [48]) with pIgG and hIgG was performed. Thirty minutes of incubation were sufficient to detect co-localisation of pIgGs along axonal fibre tracts. It should be noted that even in axonal bundles rather far from the epithelial layer, immunoreactivity against pIgG was observed. This observation is in accordance with the rather fast kinetics of intranasally applied drugs that are reported from clinical and in vivo studies (for summary see [49]). By contrast, co-localisation of hIgGs and NF200 was found only after 8 h, and here the concerned neuronal bundles were rather close to the epithelial cell layer (Figure 7A–C). Our data may indicate that an increased uptake into the *lamina propria* could be associated with an increased uptake into neuronal bundles. Thus, even if FcRn is apparently not relevant for direct uptake into neuronal fibres, an indirect connection could be plausible. However, the IgG signal observed was rather weak, which could indicate a limitation of this neuronal pathway. It should be noted however, that the immunofluorescent signal was generally weak for pIgG after 30 min, and for hIgG even after 8 h of incubation.

As described above, structures which apparently were lymphoid follicles were time-dependently spared from allogenic IgG signal. Brandtzaeg et al. described lymphoid follicles in the mucosa of the gut, the lung and the nose, consisting amongst others of B and T lymphocytes [50]. To verify that such structures were lymphoid follicles, a double staining of pIgG with either CD20 (to detect B cells, Figure 7D) or CD3 (to detect T cells, Figure 7E) was performed. In contrast to the latter study, CD20 immunoreactivity was mainly located at borders of the lymphoid follicles. For CD14 (monocytes/macrophages) the location was similar as for CD20 (data not shown). As the borders of the lymphoid follicles were not spared from pIgG signal, a strong co-localisation was observed (Figure 7D). It should be noted that the goat-anti-swine IgG secondary antibody used throughout this study does not show cross-reactivity or unspecific binding to the B cell receptor. Therefore, co-localisation of pIgG and B cells is unlikely to be caused by staining of B cells via CD20 and Fc domains of their B cell receptors. T cells were found inside the follicles; thus, no co-localisation was evident. Earlier reports suggested an endocytic uptake of IgG from dendritic cells and macrophages. Infiltrated IgGs from the mucosa may bind important antigens from the apical nasal cavity. The IgGs are proteolytically digested, including the antigen, which is subsequently presented on the cell surface to the cellular immune system. Preliminary data showed that macrophages could be detected in relatively high amounts in the subepithelial dome underneath the basal cell layer, as well as occasionally inside the follicles (data not shown). Like other antigen-presenting cells, macrophages could have engulfed and digested the pIgGs, so that they were not detectable anymore. The above described effects were not observed for xenogenic hIgG. Based on these results, an interaction of the mucosal immune system with the externally applied pIgGs could be concluded.

## 4. Discussion

N2B drug delivery is of increasing interest since it was demonstrated that tracers like wheat-germ agglutinin-HRP conjugates could be found in the axonal projections of olfactory sensory neurons and in the olfactory bulb [36]. As clinical studies with intranasally delivered CNS-active peptides and small proteins are ongoing, the delivery of larger proteins via the nasal pathway as an alternative to bypass the BBB becomes more and more attractive [13,51,52,53].

Recently, it was shown in human nasal tissue that the IgG specific transporter FcRn is expressed in the respiratory regions of the nose [19]. Here, we demonstrated that FcRn is also widely expressed in the porcine nasal mucosa, which makes the pig an interesting model for receptor-mediated uptake and transport of IgGs. We found FcRn expression in all regions of the porcine nasal mucosa, also in the *regio olfactoria*. The *regio olfactoria* is the region of interest with regard to N2B drug delivery, as the epithelium is widely spanned with olfactory sensory neurons that are projecting into the brain, but it is also innervated by the trigeminal branches that connect to the brain stem [33]. We demonstrated that in epithelial cells of the olfactory epithelium, the FcRn is strongly expressed on the apical side, whereas in blood vessels and glands a weaker basolateral expression was found. We could additionally demonstrate intracellular localisation of FcRn in the epithelial layer that hint to a possible FcRn-mediated transport mechanism. Such FcRn-mediated transcytosis has hitherto mostly been analysed in other mucosa sites in the body such as lung and gut [18,23,54,55].

The results of the IgG transport and distribution studies, double staining of IgG and FcRn together with the mentioned literature data, give strong indications for an involvement of FcRn in IgG transport through the olfactory mucosa. However, a direct FcRn-IgG interaction cannot be concluded from our co-localisation data. Further experiments using FcRn knock-out or similar models are needed to confirm the involvement of FcRn in this process. Nevertheless, our results indicate a species-preference of IgG transport mechanisms, since our data show significant differences in uptake kinetics of allogenic porcine IgGs vs. xenogenic human IgG. An alignment of human (UniProt ID P01860) and porcine (UniProt ID L8AXL3) immunoglobulin heavy constant chains and, in particular, the C_H_3 domain hosting the binding site of FcRn revealed identities of less than 50%. This is supported by Ober et al. which showed species-specific preferences by the human FcRn in vitro [28]. Their kinetic data suggested a very low affinity of human FcRn to murine IgGs, but a higher affinity to rabbit IgG. In contrast, Stirling et al. showed in vitro that hIgG yields a higher porcine FcRn-mediated uptake [17]. These results are further supported by kinetic investigations of the rabbit FcRn that showed binding to human IgG with higher affinity than rabbit IgG in surface plasmon resonance experiments [56]. Similarly, it was reported that the serum half-life of human IgGs administered to mice is significantly lower than the half-life of murine antibodies. Ober et al. (2001) showed that the recycling mechanism of FcRn was not functional with xenogenic IgGs [28]. Our data support the hypothesis of species-dependent IgG transport, as we did not observe significant hIgG levels in the *lamina propria* after 8 h of permeation. There are studies showing efficient FcRn-mediated transport of xenogenic IgGs in cell culture models that may not reflect the same conditions as under ex vivo and in vivo conditions [17,56]. However, we cannot exclude that other transport mechanisms than FcRn are responsible for our observations. For example, Ishikawa et al. found evidences that Fc gamma receptor IIb is involved in IgG transcytosis in the placental endothelium [57]. Other Fc receptors are mainly known for effector function in immune surveillance and response [58,59,60,61].

Further on, we cannot rule out the influence of the apical pH on the antibodies directly in the tissue. As it is described to be a sour environment at the apical surface aggregation of the hIgG is possible [62,63]. As we used the natural mucus layer located on the tissue explant, we do not know the pH exactly. Another possibility would be to wash the specimen and to use artificial mucus with a defined pH as described by Rinaldi et al. [63].

**Figure 8:**
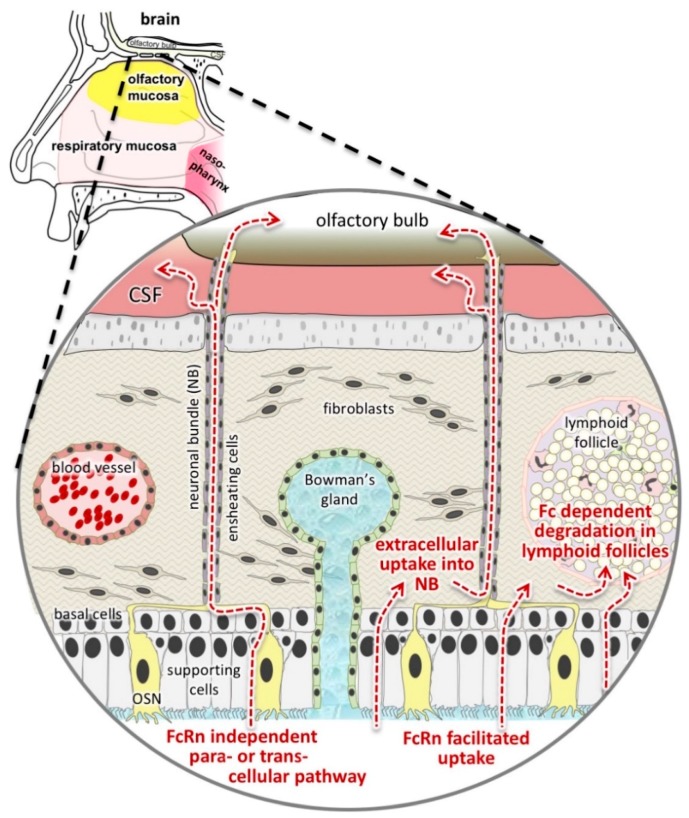
Hypotheses based on our study results: We presented evidences implying an FcRn-mediated transport in the porcine *regio olfactoria* and we have observed three different destinations of the absorbed IgGs: uptake into or along neuronal fibres (final destination: central nervous system (CNS)), uptake into blood vessels or in glands (final destination: blood and mucus), or potential degradation in lymphoid follicles (final destination: presented as antigen with a potential to cause immunogenicity). In this sketch, the uptake into blood vessels was neglected as compared to the respiratory mucosa, as the olfactory mucosa harbours only small and rare blood vessels. In upcoming studies, the potential of Fc-bearing molecules such as IgGs and Fc fusion proteins to target lymphoid follicles will be determined. In parallel, the levels of Fc-deleted proteins such as scFv or domain antibodies that reach the brain will be studied. The aim is to resolve if those pathways can be targeted separately or if intranasal N2B delivery will always be a mixture of CNS, blood, and immune system targeting. CSF: cerebrospinal fluid; Fc: Fc domain; FcRn: neonatal Fc receptor; NB: neuronal bundle; OSN: olfactory sensory neuron.

In this study, we were also interested in the involvement of neuronal fibres in N2B transport. Our results show co-localisation of neuronal fibres and pIgG after only 30 min, even in the deeper *lamina propria*. In contrast to allogenic pIgG, co-localisation of xenogenic hIgG with NF200 was only detectable after 8 h, and only close to the basal cell layer. As we did not find significant levels of hIgG in general in the *lamina propria,* this finding might be due to the concentrations used, and could be investigated in future experiments with higher concentrations for bevacizumab. But according to the most common hypothesis, intranasal applied drugs are taken up by apical dendrites of olfactory neurons and are transported inside or along the axons to the brain [21,64]. This mechanism was discussed as unspecific, and a species-dependency has not been described before. Therefore, the observation that xenogenic IgG was rarely found in or along neuronal bundles was rather unexpected. However, the olfactory ensheathing cells surrounding the neuronal fibres can be detected in the basal cell layer and in the *lamina propria* [21]. Thus, the olfactory axons are unshielded from the epithelial layer down to the basal cell layer and an uptake of molecules into the unwrapped fibre tracts starting from the basal cell layer could explain these observations (Figure 8). Finally, it can be speculated that endocytosis and allogenic FcRn-mediated transcytosis facilitate the uptake over the epithelial layer, leading to an increased absorption in neuronal bundles at the stage of the basal cells.

In general, barriers like the BBB and the blood nerve barrier protect neuronal tissues from entry of antibodies, T cells, and toxic components of the blood [65]. Previous studies revealed that the perineural route is faster than intracellular axonal transport [25]. FcRn was found to be expressed in peripheral nerves, and was further described as a transporter at the blood nerve barrier [66,67]. An uptake of IgG into neuronal bundles would indeed be surprising, as in the BBB, the FcRn is known to act as an efflux transporter, but we cannot exclude this possibility [68,69]. Another explanation may be that the uptake of IgGs into neurons occurs at the basolateral side of the epithelial cell layer or in the *lamina propria* and not by apical dendrites of the olfactory sensory neurons. Thus, the FcRn-mediated transport of allogenic IgG at the epithelial barrier could explain our observations as we demonstrated poor uptake of hIgG into the epithelial layer. In upcoming in vivo experiments, it will be of interest whether allogenic IgG can be transported into the olfactory bulb as shown for other proteins. Furthermore, the fate of the hIgG in the *lamina propria* remains to be elucidated. We observed predominantly that hIgG was co-localised with endothelial cells of cavernous bodies and blood vessels, but also in glands, although no general tissue distribution was observed in the *lamina propria*.

During the IgG distribution experiments, we found circular structures in the *lamina propria* that were almost completely spared from permeating IgG. As their morphology resembles Peyer’s patches in the gut, we started to investigate the local immune system in the *regio olfactoria*. The architecture of Peyer’s Patches in the intestine is separated into three main domains: the follicular area, the interfollicular area, and the follicle-associated epithelium [70]. Around the follicle B cells, T cells, macrophages, and dendritic cells are located in the subepithelial dome [71]. Debertin et al. found lymphoid follicles morphologically similar to Peyer’s patches in the nasal cavity of infants [34]. Similar to our findings, they discovered that lymphoid follicles are mainly located in the upper nasal cavity. Thus, morphologically the lymphoid follicles show the same features in young children and pigs.

In summary, little is known about the development and the exact function of the immune system in the upper part of the nose in mammals. Concerning the distribution of immune cells in the local lymphoid follicles, we found partially CD3^+^ cells in the germinal centre of the lymphoid follicle whereas in the subepithelial dome CD3^+^, CD20^+^, and CD14^+^ (data not shown) cells were located in high amounts. These findings are in accordance with Peyer’s patches in men and sheep [46,72,73,74,75]. Our data are somewhat in conflict with data of Brandtzaeg et al. who found mainly CD20^+^ cells inside and CD3^+^ cells outside the lymphoid follicles in human Peyer’s patches. The exact cellular composition is up to now a controversial topic. Studies show an age-related change in B- and T-cell localisation in and around lymphoid follicles [74,75].

In our uptake and distribution experiments we observed very few antibodies inside the lymphoid follicles whereas the pIgGs were concentrated in the subepithelial dome. In double staining, we saw co-localisation of CD20^+^ and pIgGs that hints to a molecular interaction. Akilesh et al. observed FcRn expression on B cells, which further strengthens our results of an interaction between permeated pIgGs with CD20^+^ cells [76]. Our results show no co-localisation of CD3^+^ cells and pIgGs. Baker et al. showed that cross-presentation of IgG immune complexes by dendritic cells is mediated by FcRn and controls cross-priming of CD8^+^ T cells in mice [77]. It is commonly known that diverse types of dendritic cells reside in the lymphoid follicle, the subepithelial dome and the surrounding epithelium in the intestine [71,72]. It is likely that the nasal lymphoid follicles show a similar distribution of dendritic cells. Double staining with CD20 and CD3 specific antibodies did not completely reveal the cellular composition in the lymphoid follicles, as most of the follicle was still unstained. Dendritic cells could be one cell type to fill the gaps.

Referring to N2B drug delivery, again the risk of undesired immunogenicity could be significant, especially when FcRn mediated processes like IgG transport are involved. Certainly, there are several links between FcRn and immune response in mucosal sites. For example, the ability of FcRn to direct antigens to inductive sites of the mucosal immune system was demonstrated to play an important role in immune surveillance [78]. It was further observed that bidirectional IgG transport retrieved luminal antigens into the mucosa, where they can be captured by dendritic cells for CD4^+^ T cell presentation [79]. An FcRn-targeted mucosal delivery of a herpes simplex virus type-2 glycoprotein fused to an Fcγ domain resulted in an effective memory immune response in mice [80].

A huge challenge and problem in investigating the clinical potential of intranasal N2B delivery of biopharmaceuticals is the choice of model organisms. Species-specific effects can be only overcome by using allogenic/surrogate proteins of the respective model species, and using the clinical candidates are of limited value. Furthermore, to elucidate potential risks of immune interactions, the differences in the organisation and morphology of nasal immune systems between species is of great importance. Rodents lack the typical nasopharynx- and nose-associated lymphoid tissue (NALT) described in human, sheep or other farm animals, but they possess locally organized lymphoid tissue at the bottom of their nasal passages [81].

Transmucosal permeation was used in several studies as a model to predict bioavailability as is well established for intestinal cellular models such as Caco-2 [82]. Also, permeation was presented in studies focusing on N2B delivery [38,40,83]. However, the data presented here raise the question about the suitability of using permeation experiments to extrapolate or conclude from these data the clinical feasibility of N2B drug delivery. Most in vitro models for permeation consist of epithelial carcinoma cells, but only some express functional FcRn [84]. The use of primary cultures seems to overcome this lack of FcRn, but here the neuronal bundles and axons as well as lymphoid structures are still missing [19,85]. As we presented in this work for olfactory mucosa, absorbed IgGs were drained either by neuronal bundles, by blood or lymphatic vessels, or by lymphoid follicles. Hence, a permeation through an in vitro model is limited in comparison with in vivo models concerning multifactorial drug pathways in vivo. Remarkably, the use of ex vivo tissue combined with histological techniques, appears to be rather close to in vivo models, but lacking of course, blood circulation and intact axonal projections. Consequently, the ex vivo nasal mucosa model complemented our test and screening battery consisting of an in vitro cellular model (RPMI 2650; [83]) and a refined intranasal administration technique in mice as in vivo model [86].

## 5. Conclusions

The present study demonstrated that functional FcRn expression in the olfactory mucosa seems to be both friend and foe for intranasal drug delivery. Our results indicate transport of allogenic IgGs across the epithelial barrier and along neuronal bundles of the olfactory sensory neurons. Whether this transport actually results in increased concentration of IgGs in the olfactory bulb and other brain regions needs to be explored in future in vivo studies.

On the other hand, our data indicate an interaction of the applied pIgGs with the local immune system, possibly a first step in a cascade causing immunogenicity against the applied drug. In further studies we will investigate the fate of proteins with and without the Fc domain when applied intranasally (see Figure 8). Probably, the immunogenicity of vaccines can be improved by fusion to Fc, while Fc-deleted antibody fragments with a lower molecular weight are better suited to reach the brain via axonal projections. In conclusion, profound investigations of transport pathways are required to develop valid pharmacokinetic models for a predictive clinical translation, but the intra-mucosal activities of drugs should not be neglected.

## Figures and Tables

**Table 1:** List of antibodies used in this study.

Antibody	Antigen	Immunogen	Host	Source, Cat. #
IgG from porcine serum	-	-	pig	Sigma-Aldrich, Germany, Cat.#I4381
bevacizumab biosimilar	VEGF	VEGF-165 isoform	humanized antibody	In-house
Anti-porcine FcRn	cytoplasmic tail of the porcine FcRn	Peptide: CPWISFHGDDVGALLPTPDLDTRMLNLRI	rabbit	Pirbright Institute, UK [17]
Anti-Neurofilament 200	neurofilament heavy polypeptide	IgG fraction of antiserum	rabbit	Sigma-Aldrich, Germany, Cat. # N4142
Anti-CD3 (SP7)	intracytoplasmic portion of the CD3 antigen	synthetic peptide: KAKAKPVTRGAGA	rabbit	NovusBio, Germany, Cat.# NB600-1441
Anti-MS4A1/CD20 (MEM-97)	CD20 (Bp35)	Raji human Burkitt’s lymphoma cell line (NM_021950.3)	mouse	NovusBio, Germany, Cat.# NBP1-44634
Anti-CD14 Monoclonal (TüK4)	CD14	not specified	mouse	Thermo Fisher Scientific, Germany, Cat.# MA5-16956
Anti-β Actin (AC-15)	β Actin	not specified	mouse	Sigma Aldrich, Germany, Cat.# A5441
Anti-murine IgG-Alexa Fluor^®^488	whole molecule mouse IgG	not specified	goat	Jackson Immuno Research Europe Ltd., UK; Cat.#115-545-003
Anti-rabbit IgG-Rhodamine Red™-X	whole molecule rabbit IgG	not specified	donkey	Jackson Immuno Research Europe Ltd., UK, Cat.#711-295-152
Anti-swine IgG-Rhodamine Red™-X	whole molecule porcine IgG	not specified	goat	Jackson Immuno Research Europe Ltd., UK, Cat.#114-295-003
Anti-human IgG-FITC (Fluorescein isothiocyanat)	whole molecule human IgG	not specified	goat	Sigma-Aldrich, Germany, Cat.# F3512
Anti-rabbit IgG-HRP	whole molecule rabbit IgG	not specified	goat	Jackson Immuno Research Europe Ltd., UK, Cat.#111-035-003
Anti-murine IgG-HRP	whole molecule mouse IgG	not specified	goat	Sigma Aldrich, Germany, Cat.# AP5278

**Table 2:** Sequences of forward and reverse primer for reverse transcriptase-PCR (RT-PCR) of the targets FcRn und β-actin.

Targets	Forward Primer (5′-3′)	Reverse Primer (5′-3′)
FcRn	CTAACAGTCAAGAGCGGCGA	AGATTCCACCATGCCAGCAA
β-actin	GACACCAGGGCGTGATGG	GCAGCTCGTAGCTCTTCTCC

## Supplementary Materials

The following are available online at http://www.mdpi.com/1999-4923/10/3/107/s1, Figure S1: Influence of damaged epithelial layer on the experimental readout.
