# Supplementary material for: Allogenic Fc Domain-Facilitated Uptake of IgG in Nasal Lamina Propria: Friend or Foe for Intranasal CNS Delivery?
Source: Pharmaceutics. 2018 Jul 26;10(3):107. doi: 10.3390/pharmaceutics10030107 (PMC6161100; doi:10.3390/pharmaceutics10030107)
Supplement: Supplementary file 1 [file pharmaceutics-10-00107-s001.pdf]

Supplementary Materials: Allogenic Fc - Domain Facilitated Uptake of IgG in Nasal Lamina Propria: Friend or Foe for Intranasal CNS Delivery?

Simone Ladel, Johannes Flamm, Arghavan Soleimani Zadeh, Dorothea Filzwieser, Julia-Christina Walter, Patrick Schlossbauer, Ralf Kinscherf, Katharina Lischka, Harald Luksch and Katharina Schindowski

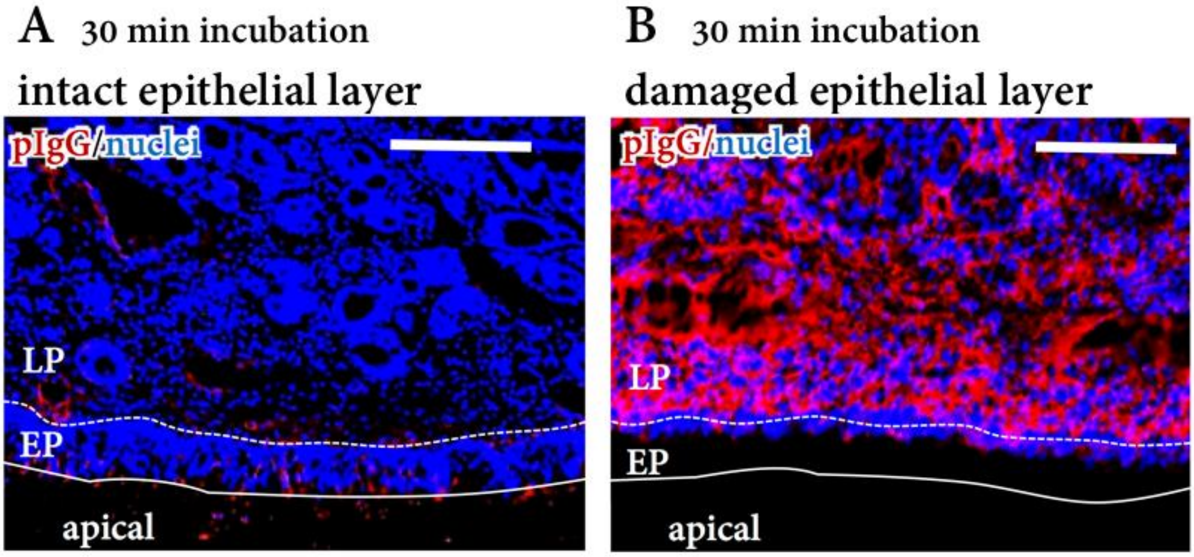

Figure S1. Influence of damaged epithelial layer on the experimental readout. (**A**) While only small amounts of exogenous IgG can cross the epithelial barrier within 30 min of incubation, (**B**) a loss of the epithelial layer that may occurs during the specimen preparation or during the experimental procedure results in a massive penetration within the same incubation time.
